# Association of cardiovascular risk factors and lifestyle behaviors with aortic aneurysm: A Mendelian randomization study

**DOI:** 10.3389/fgene.2022.925874

**Published:** 2022-08-08

**Authors:** Jiawei Zhou, Jianfeng Lin, Yuehong Zheng

**Affiliations:** ^1^ Department of Vascular Surgery, Peking Union Medical College Hospital, Chinese Academy of Medical Sciences and Peking Union Medical College, Beijing, China; ^2^ Peking Union Medical College Hospital, Chinese Academy of Medical Sciences and Peking Union Medical College, Beijing, China

**Keywords:** aortic aneurysm, diabetes mellitus, body mass index, hypertension, Mendelian randomization

## Abstract

**Objective:** To examine the causality between hypertension, diabetes, other cardiovascular risk factors, lifestyle behaviors, and the aortic aneurysm among patients of European ancestry.

**Methods:** We performed two-sample Mendelian randomization (MR) analysis to investigate the causality of 12 modifiable risk factors with aortic aneurysm, including hypertension, body mass index (BMI), waist–hip ratio (WHR), diabetes, tobacco smoking, alcohol and coffee consumption, physical activity, and sleep duration. Genome-wide significant genetic instruments (*p* < 5 × 10^–8^) for risk factors were extracted from European-descent genome-wide association studies, whereas aortic aneurysm genetic instruments were selected from the UK Biobank and FinnGen cohort. The inverse-variance weighted MR was used as the main analysis, and MR-Egger (MRE), weighted median MR, MR pleiotropy residual sum and outlier, and Phenoscanner searching were performed as sensitivity analyses. Furthermore, we calculated MRE intercept to detect pleiotropy and Cochran’s Q statistics to assess heterogeneity and conducted bidirectional MR and MR Steiger tests to exclude the possibility of reverse causality.

**Results:** We observed significantly higher risks for the aortic aneurysm in hypertension [pooled OR: 4.30 (95% CI 2.84–6.52)], BMI [OR: 1.58 (95% CI 1.37–1.81)], WHR [OR: 1.51 (95% CI 1.21–1.88)], WHR adjusted for BMI (WHRadjBMI) [OR: 1.35 (95% CI 1.12–1.63)], age of smoking initiation [OR: 1.63 (95% CI 1.18–2.26)], and tobacco use (initiation, cessation, and heaviness) [OR: 2.88 (95% CI 1.85–2.26)]. In sensitivity analysis, the causal effects of hypertension, BMI, WHRadjBMI, and tobacco use (initiation, cessation, and heaviness) remained robust.

**Conclusion:** There was a positive causal relationship between hypertension, BMI, WHR, and WHRadjBMI and aortic aneurysm.

## Introduction

Aortic aneurysms, clinically featured by permanent degradation and dilation of the aorta, are common macrovascular disorders. They could be classified according to lesion sites such as thoracic aortic aneurysm (TAA) and abdominal aortic aneurysm (AAA) (Quintana and Taylor, 2019). From previous published observational studies, several risks and protective factors for AAA have been identified, such as hypertension (Rapsomaniki et al., 2014; Kobeissi et al., 2019), history of smoking (Sode et al., 2013; Altobelli et al., 2018), body mass index (BMI) and body fat distribution (Cronin et al., 2013; Stackelberg et al., 2013), diabetes mellitus (Raffort et al., 2018), and other metabolic syndrome-related traits or lifestyles (Forsdahl et al., 2009; Kubota et al., 2018). When it comes to TAA, smoking, hypertension, and diabetes mellitus were shared (Quintana and Taylor, 2019; D’Cruz et al., 2019; Senser et al., 2021). Nevertheless, observational studies could not investigate causality because of potential confounders and reverse causation bias (Lawlor et al., 2008; Evans and Davey Smith, 2015; J ansen et al., 2014). Further research was still needed to investigate the causal role of the risk factors above, which could help better understand the mechanisms underlying aortic aneurysm development.

The Mendelian randomization (MR) design is a genetic instrumental variable analysis utilizing single nucleotide polymorphisms (SNPs) as genetic instruments to estimate the causal effect of a risk factor (exposure) on an outcome (risk for aortic aneurysm), bypassing the influence of confounding and reverse causality. Previous MR studies have investigated the causal effects of certain metabolic syndrome-related traits and lifestyle behaviors, including type 2 diabetes (T2DM) (van ’t Hof et al., 2017), BMI (Larsson et al., 2020a), smoking (Larsson et al., 2020b), and alcohol consumption (Larsson et al., 2020c) on the risk of aortic aneurysm. Tobacco smoking and alcohol consumption were considered harmful while no significant association was found between T2DM, BMI, and risk of aortic aneurysm. For other risk factors reported in the literature, including type 1 diabetes (T1DM), hypertension, and lifestyle behaviors including coffee consumption, and physical activity, the causality with risk of the aortic aneurysm has not yet been investigated, or no definitive conclusion was reached. Sleep duration was also taken into consideration because of a recent MR study that revealed a harmful effect of insomnia on intracranial aneurysms (Karhunen et al., 2021). Despite that part of the factors were already evaluated in previous studies, no relevant research systematically investigated the causal relationship between known cardiovascular risk factors and lifestyle behaviors in aortic aneurysm development. In addition, we obtained different conclusions on some factors, such as BMI.

In this study, we performed MR analysis to investigate the causal effects of 12 cardiovascular risk factors and lifestyle behaviors on the aortic aneurysm. It is hoped that this work might complement the existing evidence for the pathogenesis and primary prevention of aortic aneurysms.

## Materials and methods

### Two-sample Mendelian randomization

We performed two-sample MR analyses with the ***TwosampleMR*** package (version 0.5.6) in this study (Burgess and Thompson, 2015; Hemani et al., 2018). MR uses SNPs as genetic instruments for the causal inferences about the effect of exposure on an outcome (risk for aortic aneurysm). Genetic instruments in MR should satisfy three assumptions: (Quintana and Taylor, 2019) the SNP was associated with the exposure; (Rapsomaniki et al., 2014) the SNP was not associated with confounders that can affect the causal effect of exposure on the outcome, and (Kobeissi et al., 2019) the SNP was associated with the outcome (risk for aortic aneurysm) only through the exposure. We used publicly available data for this MR study, and the access to these data was described in each of the subsequent GWASs (Table 1).

**TABLE 1:** Overview of the data sources of the instrumental variables used in the MR study (UK Biobank/FinnGen).

Risk factors	SNP	SNP available	Outliers^a^	Potential confounders^b^	Sample size	Ancestry	Units	PVE	Overlap^c^
Hypertension Elsworth et al. (2019)	216	210/204	0/0	55/54	4,62,933	European	1 SD increase in hypertension	2.8%/2.8%	60%–70%/none
BMI Yengo et al. (2018)	490	477/467	0/2	177/173	6,81,275	European	1 SD increase in BMI	4.8%/4.7%	85%/none
WHR Pulit et al. (2019)	206	202/199	1/2	129/127	6,94,649	European	1 SD increase in WHR	2.2%/2.2%	85%/none
WHR adjusted for BMI Pulit et al. (2019)	206	202/198	1/2	64/65	6,97,734	European	1 SD increase in WHR adjusted for BMI	2.8%/2.8%	87%/none
T2DM Xue et al. (2018)	118	115/114	0/0	50/49	6,55,666	European	Odds of T2DM	13.9%/13.8%	79%/none
T1DM Onengut-Gumuscu et al. (2015)	36	36/36	0/0	4/4	29,652	European	Odds of T1DM	17.9%/17.9%	None/none
Age of smoking initiation Liu et al. (2019)	202	194/193	0/0	45/44	12,32,091	European	Ever smoked regularly compared with never smoked	0.6%/0.6%	≈30%–35%/none
Tobacco use (initiation, cessation, and heaviness) Wootton et al. (2020)	124	118/116	0/0	39/39	4,62,690	European	1 SD increase in CSI (comprehensive smoking index)	1.1%/1.1%	Full/none
Alcohol consumption Liu et al. (2019)	71	68/68	0/0	25/25	9,41,280	European	1 SD increase in log transformed alcoholic drinks/week	0.5%/0.5%	≈30%–35%/none
Coffee consumption Cornelis et al. (2015)	4	4/4	0/0	3/3	1,21,824	Mixed	1 cup increase of coffee consumed/day	0.5%/0.5%	None/none
Physical activity Klimentidis et al. (2018)	19	19/19	0/1	4/4	3,77,234	European	1 SD increase in moderate to vigorous physical activity (MVPA)/MET-min/week	0.2%/0.2%	Full/none
Sleep duration Doherty et al. (2018)	12	12/12	0/0	3/3	91,105	European	1 SD increase in sleep duration	0.5%/0.5%	Full/none

a Outliers: outlier SNPs detected by MR-PRESSO (UK Biobank/FinnGen).

b Potential Confounders: SNPs associated with other risk factors of aortic aneurysm (UK Biobank/FinnGen) reported in previous GWAS using Phenoscanner.

c Overlap: The estimated overlap of UK Biobank/FinnGen with the risk factor GWASs.

Abbreviations: BMI, body mass index; PVE, proportion of variance explained; SD, standard deviation; SNP, single nucleotide polymorphism; T1DM, type 1 diabetes; T2DM, type 2 diabetes; WHR, waist–hip ratio.

### Exposure

Based on previous studies, we selected two categories of phenotypes that have been associated with aortic aneurysm: (Quintana and Taylor, 2019) metabolic syndrome-related traits: diabetes (both T2DM and T1DM), and several other metabolic syndrome-related traits, including hypertension, BMI, waist–hip ratio (WHR), and WHR adjusted for BMI (WHRadjBMI); and (Rapsomaniki et al., 2014) lifestyle behaviors: age of smoking initiation, tobacco use (initiation, cessation, and heaviness), alcohol consumption, coffee consumption, physical activity, and sleep duration.

We used genome-wide association studies (GWASs) with large sample sizes and cohorts completely or mainly composed of individuals of European ancestry as data sources for the genetic instruments of the phenotypes. First, we included SNPs associated with each selected trait at the genome-wide significance threshold (*p* < 5 × 10^–8^). If linkage disequilibrium (LD) was present (*r* ^2^ > 0.001), we only used the SNP with the strongest association to ensure the independence assumption. We also calculated the F statistics for each SNP and used only SNP with F statistics larger than 10 to avoid weak instrument bias (Burgess and Thompson, 2011). Then, for any unavailable SNPs in GWAS for aortic aneurysms, we used SNPs with LD of at least *r* ^2^ > 0.80 as an alternative (Peters et al., 2021). At last, we included 210/204 SNPs (UK Biobank/FinnGen) for hypertension (Elsworth et al., 2019), 477/467 for BMI (Yengo et al., 2018), 202/199 for WHR (Pulit et al., 2019), 202/198 for WHRadjBMI (Pulit et al., 2019), 115/114 for type 2 diabetes (Xue et al., 2018), 36/36 for type 1 diabetes (Onengut-Gumuscu et al., 2015), 194/193 for age of smoking initiation (Liu et al., 2019), 118/116 for tobacco use (initiation, cessation, and heaviness) (Wootton et al., 2020), 68/68 for alcohol consumption (Liu et al., 2019), 4/4 for coffee consumption (Cornelis et al., 2015), 19/19 for physical activity (Klimentidis et al., 2018), and 12/12 for sleep duration (Doherty et al., 2018) (Table 1; Supplementary Table S1). We calculated the proportion of the explained variance (PVE) of the exposure by the genetic instrument using an already published formula PVE = beta^2^/(beta^2^ + N*se^2^) (Shim et al., 2015), where beta is the effect size, se is the standard error, and N is the sample size of each instrument. We then summed them up to represent the proportion of variance explained by all SNPs used, which ranged from 0.2% for physical activity to 17.9% for T1DM.

### Outcome selection

We obtained GWAS summary statistics of aortic aneurysms from two publicly available cohorts, namely, the UK Biobank and the FinnGen cohorts. The UK Biobank is a UK cohort study for the general population, including 1,374 aortic aneurysms (Phecode 442.1) patients and 4,00,595 controls until 2017 (Sudlow et al., 2015). The GWAS in UK Biobank aortic aneurysm was conducted with SAIGE on 28 million imputed variants (Zhou et al., 2018). The FinnGen study uses Finnish nationwide cohorts and biobanks and then combines genomic information, including aortic aneurysm status. GWAS summary statistics estimated with the SAIGE algorithm were obtained from the FinnGen R6 release, including 3,658 individuals with aortic aneurysms and 2,44,907 controls, with 1,69,62,023 genotyped SNPs (FinnGen, 2022). Sample overlapping in several exposure GWASs with aortic aneurysms was substantial in the UK Biobank cohort but very limited in the FinnGen cohort (Table 1). We also calculated the power using a web-based application (https://sb452.shinyapps.io/power/) (Burgess, 2014; FinnGen, 2022).

### Statistical analysis

We used the inverse-variance weighted (IVW) method for the main analyses under the random-effects model for each trait. The IVW method combines the Wald ratio estimates of each SNP (the beta coefficients for the SNP-aortic aneurysm association divided by the beta-coefficient for the SNP-exposure association) and calculates the weighted average of the Wald ratio estimates as the causal estimate. The IVW is the most commonly used and has the greatest statistical power but might be biased when the assumptions of MR were violated (Burgess et al., 2013). The UK Biobank and FinnGen cohort estimates were then pooled with a fixed-effect meta-analysis.

We then conducted several sensitivity analyses. First, we utilized the MR-Egger (MRE) regression to assess directional pleiotropy. The MRE method uses the average pleiotropic effects as the intercept but is less efficient and sensitive to outliers (Bowden et al., 2015). Second, we used the weighted median (WM) approach as sensitivity analysis, which provides valid estimates when more than half of the SNPs satisfy the instrumental variable assumptions. Third, we applied the MR pleiotropy residual sum and outlier (MR-PRESSO) method to exclude outlier SNPs (*p* < 0.10) that are potentially horizontally pleiotropic and to check whether the causal estimate changed with the exclusion of the outlying SNPs. Fourth, we performed a look-up of previously reported genome-wide significant association *via* R ***phenoscanner*** packages for any SNPs used (Staley et al., 2016; Kamat et al., 2019). The association was considered as potential pleiotropy and documented when satisfying the following criteria: (Quintana and Taylor, 2019) the association was genome-wide significant (*p* < 5 × 10^–8^); (Rapsomaniki et al., 2014) the SNPs associated with either lipid metabolism or any exposure explored in our study; and (Kobeissi et al., 2019) the GWAS was conducted in a population of European ancestry. Then, we excluded identified pleiotropic SNPs and performed MR IVW again to test the robustness of the causal effects.

Several additional tests were conducted to detect pleiotropy, including the MRE intercept (Burgess and Thompson, 2017) and Cochran’s Q statistics of MR IVW (Greco et al., 2015), to assess between-instrument heterogeneity. We also performed bidirectional MR (Davey Smith and Hemani, 2014) and MR Steiger tests (Hemani et al., 2017) to exclude the possibility of reverse causality. For bidirectional MR, genetic instruments were selected from the largest GWAS to date for aortic aneurysms following the same inclusion criteria described above. We also performed bidirectional MR (Davey Smith and Hemani, 2014) and MR Steiger tests (Hemani et al., 2017) to exclude the possibility of reverse causality. For bidirectional MR, genetic instruments were selected from the largest GWAS to date for aortic aneurysms following the same inclusion criteria described above (Klarin et al., 2020). Since summary statistics for coffee consumption GWAS were unavailable (Cornelis et al., 2015), we used “filtered coffee intake” reported in UK Biobank as an alternative (Elsworth et al., 2019). The remaining 11 risk factors for aortic aneurysms used the same GWASs mentioned above (Onengut-Gumuscu et al., 2015; Doherty et al., 2018; Klimentidis et al., 2018; Xue et al., 2018; Yengo et al., 2018; Elsworth et al., 2019; Liu et al., 2019; Pulit et al., 2019; Wootton et al., 2020). At last, we performed two-sample MR on each SNP individually and leave-one-out analyses.

All statistical tests are two-tailed, and a *p*-value smaller than 0.05 was considered statistically significant. To account for multiple testing in our primary analyses of aortic aneurysms concerning the 12 risk factors, we used the Benjamini–Hochberg method to calculate a multiple testing–adjusted *p*-value (Benjamini and Hochberg, 1995). All the statistical analyses were conducted using R (version 4.0.5).

## Results

### Metabolic syndrome and lifestyle-related risk factors and aortic aneurysm: Main results

**FIGURE 1:**
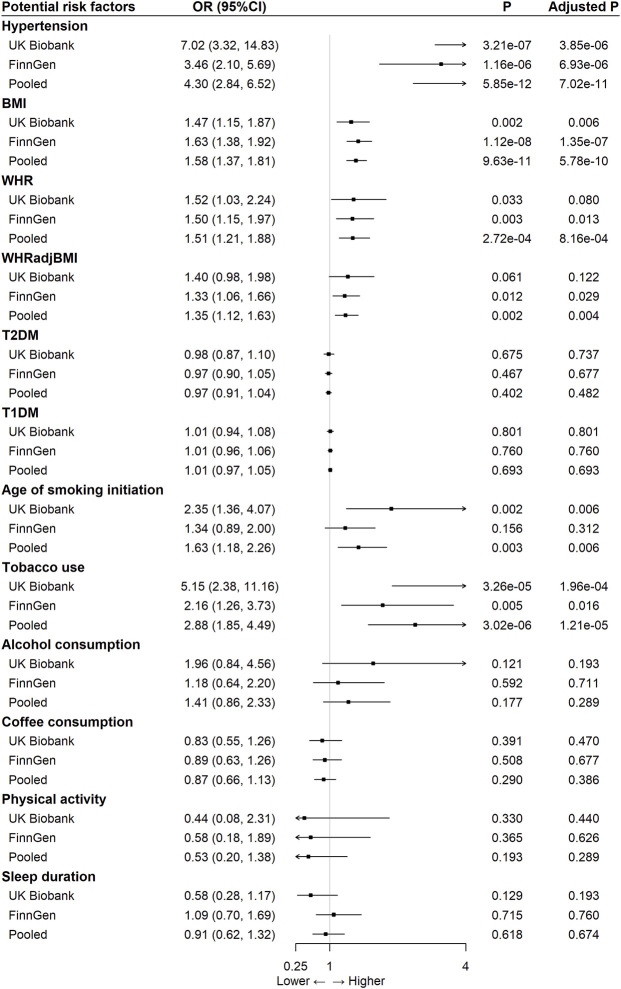
The association between 12 modifiable risk factors and aortic aneurysm using the inverse-variance weighted method.

In the pooled analysis, we observed significant causal effects of following modifiable risk factors on aortic aneurysm (Figure 1): hypertension [pooled OR: 4.30 (95% CI 2.84–6.52), *p*-adjusted = 7.02 × 10^–11^], BMI [OR per one SD increase: 1.58 (95% CI 1.37–1.81), *p*-adjusted = 5.78 × 10^–10^], WHR [OR per one SD increase: 1.51 (95% CI 1.21–1.88), *p*-adjusted = 8.16 × 10^–4^], WHRadjBMI [OR per one SD increase: 1.35 (95% CI 1.12–1.63), *p*-adjusted = 0.004], age of smoking initiation [OR: 1.63 (95% CI 1.18–2.26), *p*-adjusted = 0.006], and tobacco use (initiation, cessation, and heaviness) [OR: 2.88 (95% CI 1.85–2.26), *p*-adjusted = 1.21 × 10^–5^]. No significant causal effects were observed for T2DM, T1DM, alcohol consumption, coffee consumption, physical activity, and sleep duration (Figure 1). In addition, there were no obvious differences between the causal effects estimated from UK Biobank and FinnGen for any risk factors explored. For pooled analysis, only five risk factors, namely, hypertension, BMI, WHR, WHRadjBMI, and tobacco use, had at least 75% power to reveal statistically significant causal relationships (Supplementary Table S2).

### Robustness of the results

Several alternative MR algorithms were further performed, including MR-Egger, WM MR, and MR-PRESSO. MR-Egger method attenuated the causal effects of the following risk factors on aortic aneurysms: hypertension, WHR, and age of smoking initiation (Supplementary Figures S1, S2). The results of the WM approach revealed significant causal relationships between hypertension, BMI, WHRadjBMI, and tobacco use (initiation, cessation, and heaviness) with aortic aneurysm but not for WHR and age of smoking initiation as MR IVW (Supplementary Figures S1, S3). The MR-PRESSO revealed 0/2 (for UK Biobank/FinnGen, respectively) outlier SNPs for BMI, 1/2 outlier SNPs for WHR, 1/2 outlier SNPs for WHRadjBMI, and 0/1 outlier SNP for physical activity (Table 1). Outlier correction did not substantially change the OR estimates for BMI, WHR, WHRadjBMI, and physical activity (Supplementary Figures S1, S4).

We also used ***phenoscanner*** to identify SNPs associated with confounders for the causal effects of exposure on outcome. We reported 55/54 (for UK Biobank/FinnGen, respectively) SNPs for hypertension, 177/173 SNPs for BMI, 129/127 SNPs for WHR, 64/65 SNPs for WHRadjBMI, 50/49 SNPs for T2DM, 4/4 SNPs for T1DM, 45/44 SNPs for age of smoking initiation, 39/39 SNPs for tobacco use (initiation, cessation, and heaviness), 25/25 SNPs for alcohol consumption, 3/3 SNPs for coffee consumption, 4/4 SNPs for physical activity, and 3/3 SNPs for sleep duration, in order to have pleiotropic effects on confounders, including lipid metabolism (Table 1; Supplementary Table S1). After excluding the pleiotropic SNPs, MR IVW analysis indicated significant causal effects on aortic aneurysm of hypertension, BMI, and tobacco use (initiation, cessation, and heaviness) as shown in Supplementary Figures S1, S5.

The MR-Egger regression findings suggested potential pleiotropy for hypertension and alcohol consumption in UK Biobank-based analysis and potential pleiotropy for T1DM in the FinnGen cohort, as the intercept were all significantly not equal to 0 (*p* = 0.025, 0.040, and 0.041, respectively). The Cochran’s Q statistic with the IVW method revealed significant heterogeneity across SNPs used in the analysis for WHR and WHRadjBMI based on UK Biobank GWAS and in the analysis using FinnGen cohort data for BMI, WHR, WHRadjBMI, age of smoking initiation, and physical activity (Supplementary Table S3).

Reverse causality was explored by the bidirectional MR and MR Steiger tests. Bidirectional MR suggested a noncausal relationship between the aortic aneurysm on any of the 12 risk factors after multiple comparisons (Supplementary Table S4). As for the MR Steiger test to orient the causal direction between the exposure and the risk for aortic aneurysm, we found that there were strong pieces of evidence for hypertension, BMI, WHR, WHRadjBMI, age of smoking initiation, tobacco use (initiation, cessation, and heaviness), and physical activity as the causal risk factors for aortic aneurysm when using UK Biobank data. However, when focused on the FinnGen cohort, only age of smoking initiation and physical activity did not pass the MR Steiger test (Supplementary Table S5).

At last, MR on each SNP individually and leave-one-out sensitivity analysis in UK Biobank and FinnGen cohorts, respectively, were reported in Supplementary Tables S6–S9.

## Discussion

Our MR study confirmed a robust causal relationship between several modifiable risk factors and aortic aneurysm for the first time, including hypertension, BMI, WHRadjBMI, and tobacco use (initiation, cessation, and heaviness). In addition, no reversed causation was detected in all investigated factors. Furthermore, we identified WHR and age of smoking initiation as potential causes for aortic aneurysms, but the result varied across different MR algorithms. However, our study did not reveal the significant causal effects of T1DM, alcohol consumption, coffee consumption, physical activity, and sleep duration on the aortic aneurysm in the population of European ancestry.

The relationship between hypertension, BMI, WHRadjBMI, and tobacco use (initiation, cessation, and heaviness) and aortic aneurysm was likely to be causal. The causal relationships were consistent in the main analysis and with a maximum of one failure among the other four sensitivity tests. We noticed that MR-Egger tended to fail more frequently than the other algorithms. The observed failure might suggest outliers or violations of the inside assumptions in the cases of hypertension and age of smoking initiation (Bowden et al., 2015). Among the four risk factors mentioned above, the nonsignificant result after Phenoscanner search and exclusion was observed in WHRadjBMI. The failure might occur because of pleiotropy of the SNPs since 64/65 SNPs used in the main analysis were identified to have associations with traits beyond WHRadjBMI. We also observed a significant Cochran’s Q statistic for WHRadjBMI, suggesting potential pleiotropy. However, for WHR and age of smoking initiation, there were apparent inconsistencies among the main analysis and sensitivity analyses. More than half of the SNPs as genetic instruments for WHR were associated with other phenotypes. Similar to WHRadjBMI, the significant effect of WHR on aortic aneurysm was abolished after Phenoscanner exclusion and Cochran’s Q statistic revealed pleiotropy. MR-Egger intercepts and Cochran’s Q statistic did not report pleiotropy and heterogeneity regarding age of smoking initiation. However, the proportion of potential invalid SNPs for age of smoking initiation was approximately one-quarter, and no significant results were observed after Phenoscanner exclusion.

Hypertension was a widely recognized risk factor in aortic aneurysm development. Although several epidemiological studies revealed a positive correlation between hypertension and aortic aneurysm, no causal relationships have been reported in previous MR studies (Vardulaki et al., 2000; Kobeissi et al., 2019; Mori et al., 2020). Our study identified the forward causation between hypertension and aortic aneurysm (*p*-adjusted < 0.001) for the first time. In a meta-analysis enrolling 14 cohort studies with a total of 26,943 cases and 5,317,552 participants, the overall RR to develop AAA in hypertensive patients was 1.66 (95% CI: 1.49–1.85) (*p* < 0.001) compared to patients without hypertension (Kobeissi et al., 2019). In addition to AAA, a cross-sectional study on computed tomographic scans of 21,295 patients investigated the association between atherosclerotic risk factors and ascending TAA (ATAA). The multivariate analysis results indicated that hypertension was positively associated with ATAA ≥ 4.5 cm (OR: 2.08; 95% CI: 1.44–3.03; *p* < 0.001). On this basis, we further suggested that hypertension could mediate the susceptibility to aortic aneurysms.

As a critical metabolic factor, several conventional observational studies have investigated the role of BMI in aortic aneurysm development (Cronin et al., 2013; Stackelberg et al., 2013). Our MR study revealed forward causation between indicators for the relative weight and body fat distribution (BMI, WHR, and WHRadjBMI) and aortic aneurysm (*p*-adjusted < 0.005). The results coincided with a previous meta-analysis in which three studies, involving 305,726 participants, found a positive association between BMI and AAA. Moreover, a cross-sectional study of 12,203 men in Western Australia identified a greater waist–hip ratio as an independent and positive risk factor associated with AAA prevalence. However, considering the association between WHRadjBMI and aortic aneurysm, opposite conclusions were acquired in a previous MR study in the Dutch cohort, which might be related to the study population (van 't Hof et al., 2017). Given the lack of relevant cohort studies, the WHRadjBMI causal effects on aortic aneurysms still needed more investigations.

The causal effect of smoking on aortic aneurysms has been long recognized. The aortic aneurysm was the cardiovascular disease most strongly associated with smoking (Pujades-Rodriguez et al., 2015). Current smoking was the only modifiable risk factor for AAA growth in a meta-analysis involving 15,475 patients (Ulug et al., 2016). Previous MR analysis also suggested a causal relationship between tobacco use (initiation, cessation, and heaviness) and AAA, which is consistent with our findings. However, the same study also reported a significant role in age of smoking initiation (Larsson et al., 2020b). The discrepancy, as suggested by Phenoscanner searching and exclusion, might be because nearly one-quarter of the genetic instruments used for age of smoking initiation were invalid.

Last but not least, we performed an MR study on the causal relationship between diabetes and aortic aneurysm. In a cohort study involving 1.9 million participants to explore the association between T2DM and AAA, T2DM was negatively associated with AAA with a median follow-up of 5.5 years (OR = 0.46, 95% CI: 0.35–0.59, *p* < 0.0001) (Shah et al., 2015). A Spanish national retrospective study involving 1,15,020 patients admitted with AAA suggested that the incidences of AAA were significantly higher among nondiabetic elders (age > 70 years old) (*p* < 0.05) (Lopez-de-Andrés et al., 2015). Another population-based prospective study also reported the protective role of T2DM in AAA development (OR = 0.57, 95% CI: 0.40–0.82) (Larsson et al., 2018). However, our analysis did not reveal statistically significant causal effects for both T1DM and T2DM. The failure might be because of the lacking of statistical power of our study as suggested using power analysis. Nonetheless, we cannot exclude the possibility that previous observational studies were confounded. Further well-designed aortic aneurysm GWASs might facilitate the MR analysis on the effects of diabetes on the risk of aortic aneurysm.

## Strengths and limitations

A key strength of our MR study was that we utilized the two independent aortic aneurysm GWASs with large sample sizes to investigate the causal effects of various relevant risk factors on aortic aneurysms. We also conducted multiple sensitivity analyses, pleiotropy detection, and reverse causality tests to carefully draw a conclusion and fulfill MR design’s advantages of minimizing residual confounders.

Our study has several limitations. First, we only focused on European ancestry, and analyses were performed with combined data for males and females. The generalizability of the results to non-European populations and certain sex was limited. Second, the overlap between the population of exposure and outcome was inevitable, especially for UK Biobank, which could cause a shift of error toward the observational study. Third, the small variance explained by using the genetic instruments for several risk factors might result in insufficient statistical power to draw a powerful conclusion. Our power analysis also suggested lacking statistical power. It might be because the UK Biobank and FinnGen cohorts utilized in the present study were not case–control designs, which meant the detection of a particular disease might be insufficient. Fourth, horizontal pleiotropy was present and might introduce bias from the unexpected association between SNPs and potential confounders through relevant tests that were already conducted. Last but not least, some practical differences do exist between AAA and TAA. Further relevant studies might be needed to explore the difference..

## Conclusion

In conclusion, we identified a positive causal relationship between hypertension, BMI, WHRadjBMI, and tobacco use (initiation, cessation, and heaviness) and aortic aneurysm. The potential harmful effect of WHR and age of smoking initiation could be further explored. Our study might contribute to the understanding of aortic aneurysm etiology and management of population health. In addition, the negative result of T2DM on the risk of aortic aneurysm indicated that some confounding factors contributed to the protective role of T2DM in observational studies.

## Data Availability

The original contributions presented in the study are included in the article/Supplementary Material; further inquiries can be directed to the corresponding author.

## Supplementary material

Supplementary Table S1 Single nucleotide polymorphisms (SNPs) used as genetic instruments for Mendelian randomization.

Supplementary Table S2 xxxxxxx

Supplementary Table S3 Pleiotropy test for SNPs used for 12 risk factors for aortic aneurysm.

Supplementary Table S4 Bidirectional MR tests for the role of aortic aneurysm role 12 risk factors.

Supplementary Table S5 Mendelian randomization (MR) Steiger tests for orientation of the association between 12 risk factors and aortic aneurysm.

Supplementary Table S6 Individual SNP MR on 12 risk factors and aortic aneurysm in the UK Biobank.

Supplementary Table S7 Individual SNP MR on 12 risk factors and aortic aneurysm in FinnGen cohort.

Supplementary Table S8 Leave-one-out MR analysis on 12 risk factors and aortic aneurysm in UK Biobank.

Supplementary Table S9 Leave-one-out MR analysis on 12 risk factors and aortic aneurysm in FinnGen cohort.

Supplementary Figure S1 xxxx

Supplementary Figure S2 Association between 12 modifiable risk factors and aortic aneurysm using the MR-Egger regression method.

Supplementary Figure S3 Association between 12 modifiable risk factors and aortic aneurysm using the weighted median method.

Supplementary Figure S4 Association between 12 modifiable risk factors and aortic aneurysm using the MR-PRESSO method.

Supplementary Figure S5 Association between 12 modifiable risk factors and aortic aneurysm using the IVW method after phenoscanner exclusion of pleiotropic SNPs.
