# Supplementary figures and images for: Association of cardiovascular risk factors and lifestyle behaviors with aortic aneurysm: A Mendelian randomization study
Source: Front Genet. 2022 Aug 8;13:925874. doi: 10.3389/fgene.2022.925874 (PMC9393757; doi:10.3389/fgene.2022.925874)

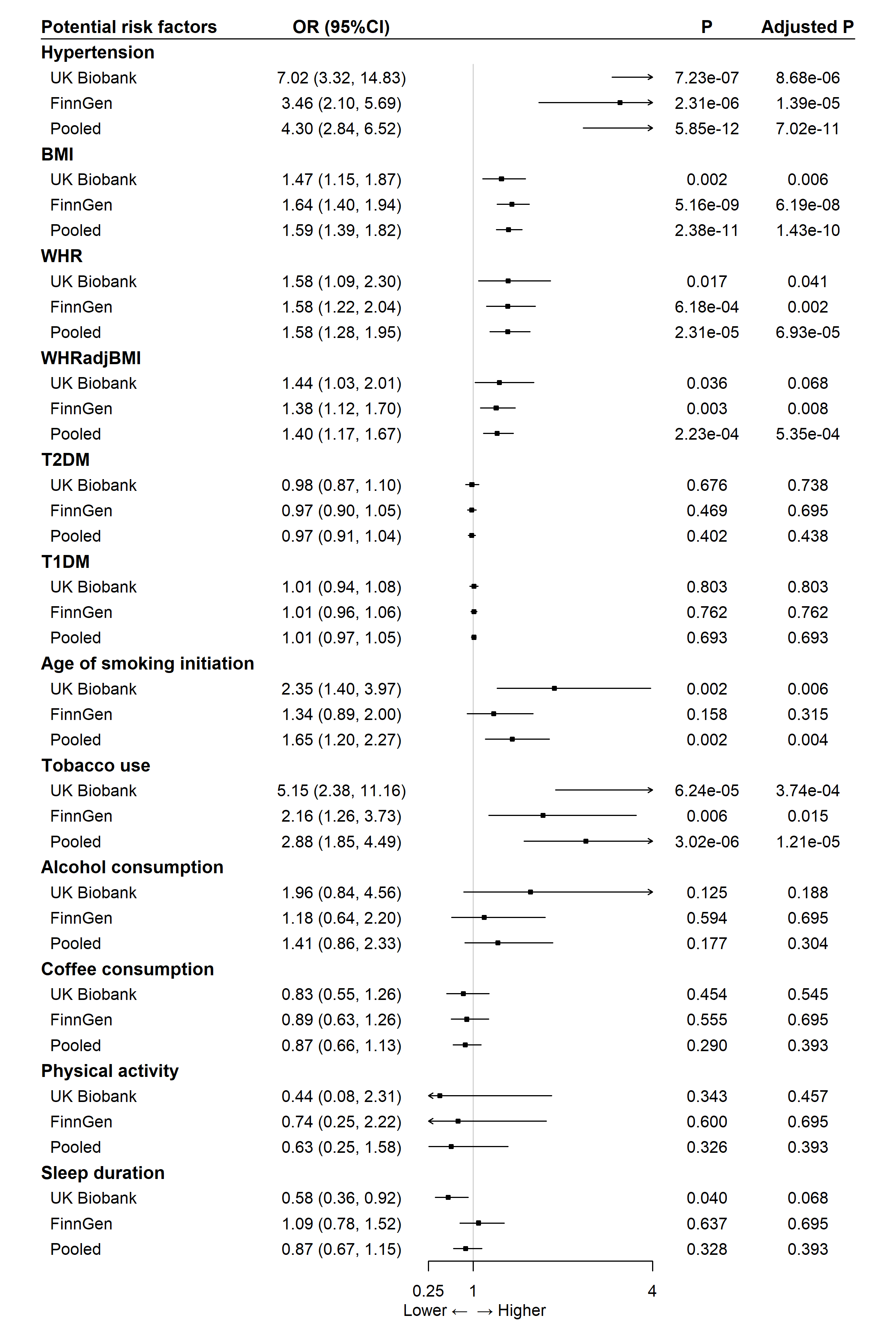

Supplement: Supplementary file 4 [file Image3.TIF]

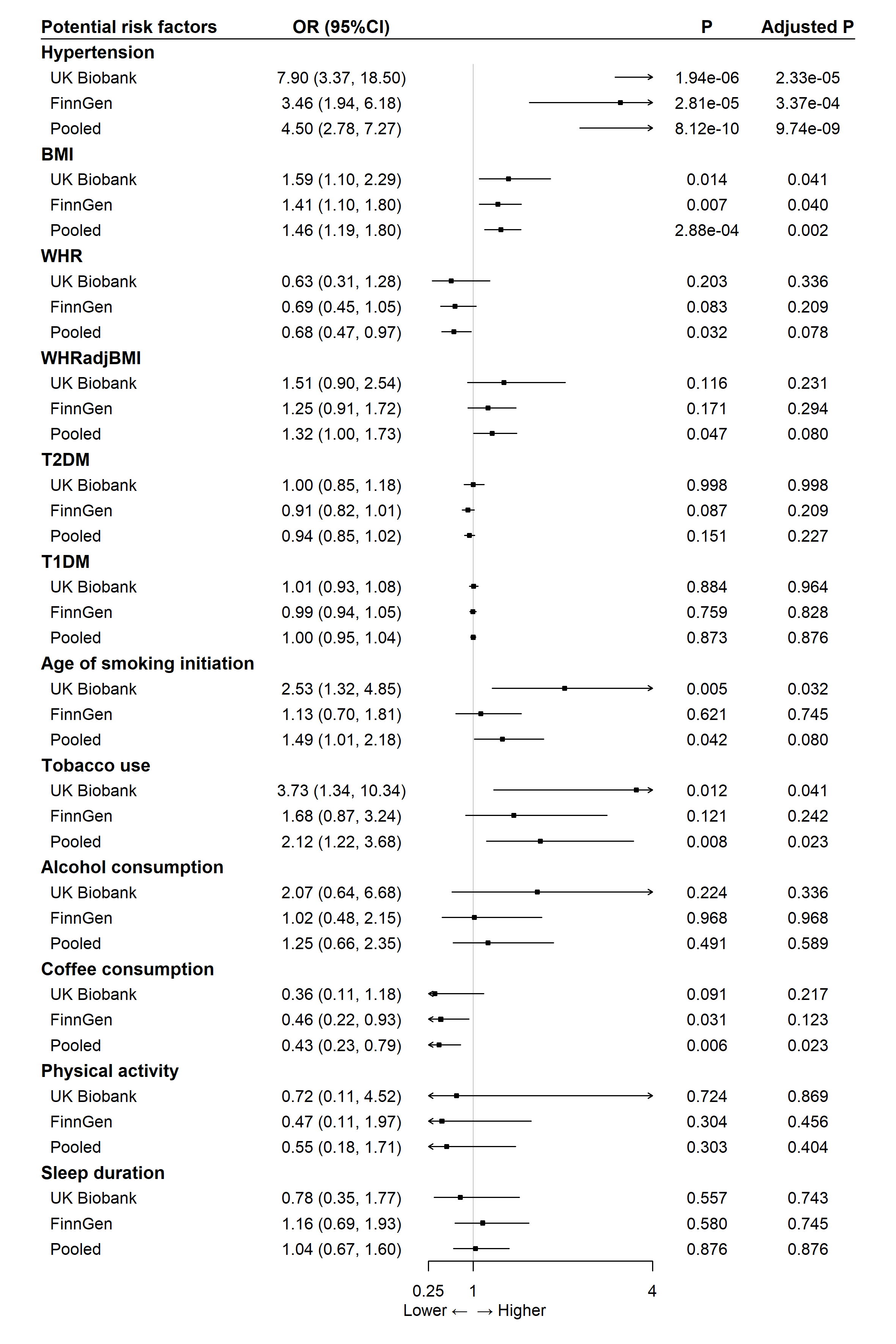

Supplement: Supplementary file 5 [file Image4.TIF]

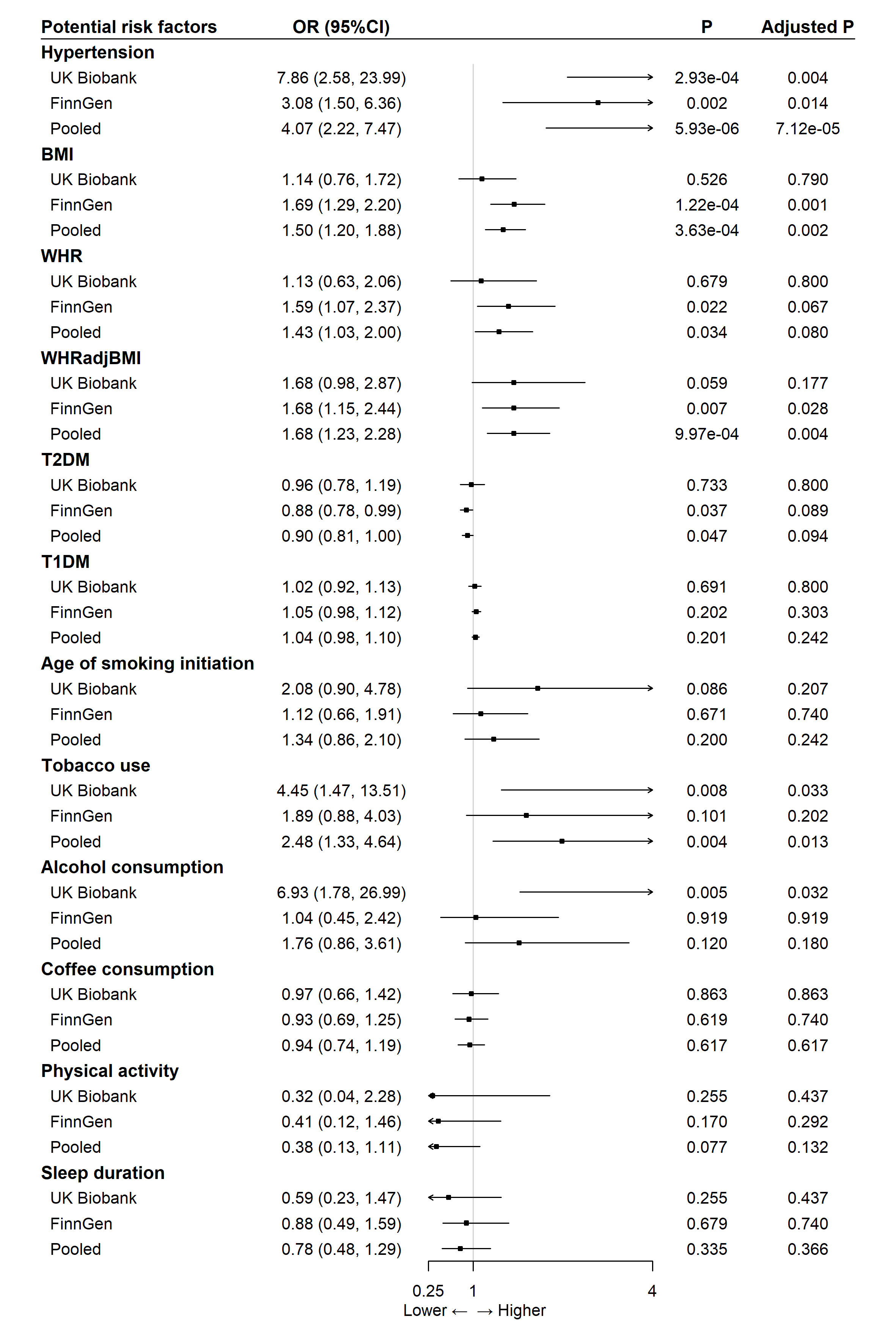

Supplement: Supplementary file 6 [file Image2.TIF]

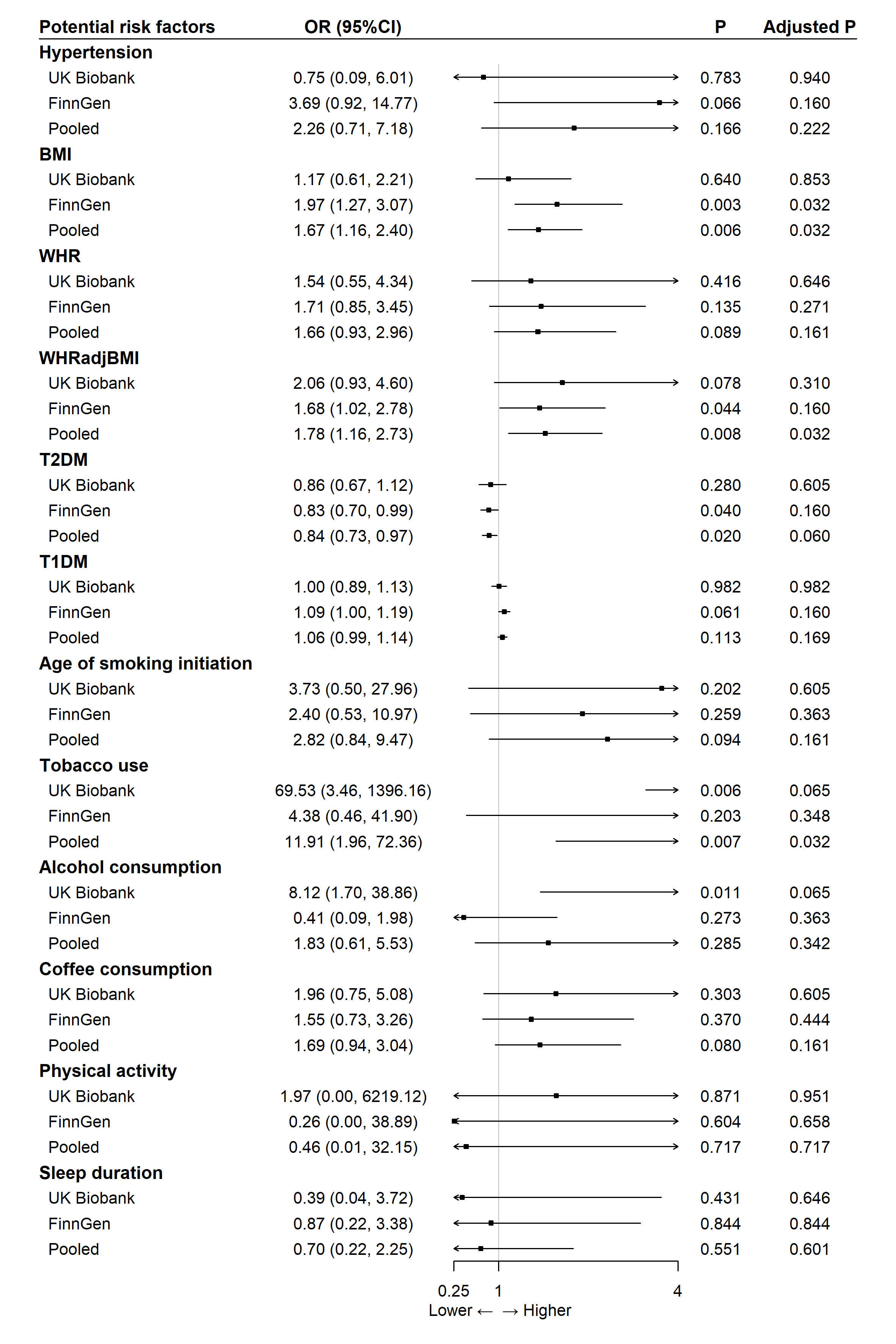

Supplement: Supplementary file 7 [file Image1.TIF]

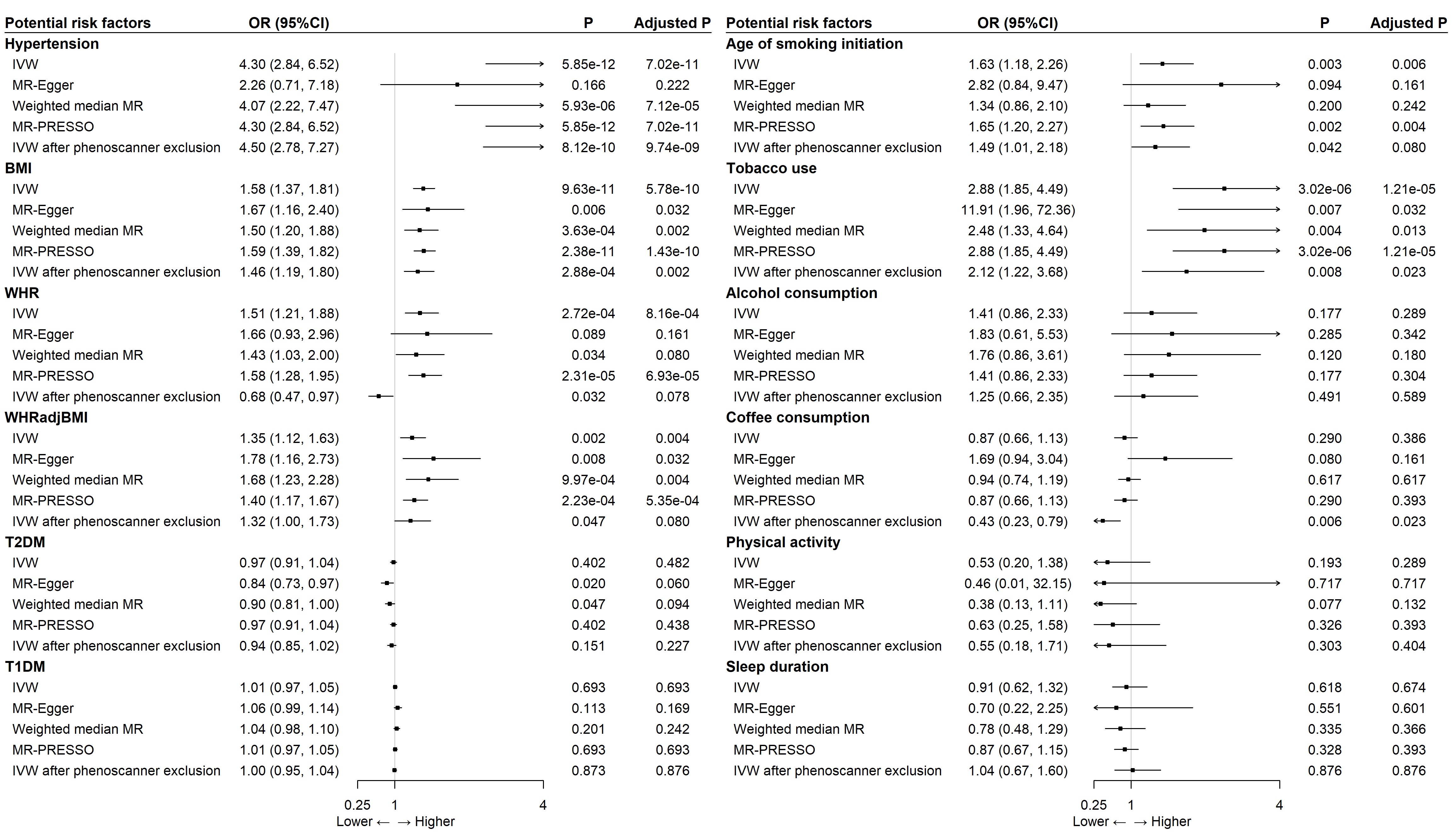

Supplement: Supplementary file 8 [file Image5.PNG]
